# MiR-134 regulates the proliferation and invasion of glioblastoma cells by reducing Nanog expression

**DOI:** 10.3892/ijo.2013.1844

**Published:** 2013-03-04

**Authors:** CHAO SHI NIU, YANG YANG, CHUAN-DONG CHENG

**Affiliations:** 1 Department of Neurosurgery, Anhui Provincial Hospital Affiliated to Anhui Medical University, Hefei, Anhui 230001, P.R. China; 2 Anhui Province Key Laboratory of Brain Function and Brain Disease, Hefei, Anhui 230001, P.R. China; 3 Anhui Provincial Stereotactic Neurosurgical Institute, Hefei, Anhui 230001, P.R. China

**Keywords:** glioma, miR-134, Nanog

## Abstract

MiR-134 is a brain-enriched miRNA that plays an essential role in the development of the embryonic stem cell-orientated differentiation to central nervous system by suppression of Nanog and neural development (including neurons, cylindraxile and dendrites) and has been shown to be downregulated in oligodendrogliomas (ODG) and glioblastomas (GBM), suggesting its possible involvement in brain tumor progression. In this study, we defined the expression and function of miR-134, which we found to be downregulated in glioma samples and the glioblastoma cell line U87 by SYBR green real-time quantitative reverse transcription-PCR (real-time PCR). Early reports have characterized Nanog as a direct target of miR-134 by a dual-luciferase reporter assay in 293T cells. In our study, overexpression of miR-134 in U87 glioblastoma cells resulted in significant downregulation of Nanog mRNA levels as well as protein levels. miR-134 overexpression reduced the proliferation, invasiveness and migration capability of U87 cells while promoted apoptosis of these cells *in vitro* and suppressed the growth of tumor xenografts *in vivo*. These findings demonstrated that miR-134 deregulation is common in human gliomas. Restoration of its function inhibits cell proliferation, invasion and migration capability and promotes apoptosis, which could be partly due to its inhibitory effect on Nanog protein expression in glioblastoma cells. MiR-134 could play an important role as a tumor suppressor relying on its direct translational attenuation of Nanog.

## Introduction

Gliomas are the most common malignant primary brain tumors. Despite aggressive surgery, radiation and chemotherapy, the median survival is only 12–15 months for glioblastoma multiforme (GBM) (1). Therefore, it is crucial to investigate the mechanism involved in the development and progression of glioma and to find new therapeutic targets.

MicroRNAs are small, endogenous, nonprotein-coding RNA molecules which have a functional role as negative gene regulators through complementarity to the 3′-untranslated region (3′-UTR) of mRNAs (2), it plays critical regulatory roles in the diverse biological processes of development, differentiation and apoptosis. MiRNAs show pleiotropic and pivotal effects on a variety of pathological and physiological cellular processes (3,4). Furthermore, each miRNA can potentially regulate hundreds of mRNAs and more than one-third of human genes might be miRNA targets (5). A considerable amount of evidence indicates that miRNAs are involved in human cancer (6,7). Deregulated miRNAs exhibit oncogenic or tumor suppressor properties. However, only a small fraction of miRNAs has already been investigated. In glioma, apart from upregulated miRNAs such as miR-21, the functions of downregulated miRNAs are relatively unknown.

As a stemness gene, Nanog has been reported to be significant in ESCs (embryonic stem cells), in which the level and activity are the pluripotent status indicators of ESCs (8). Nanog, a transcription factor, is not only known to play an essential role in ESC maintenance and differentiation by reprogramming gene sets that include OCT4, SOX2, c-myc and Klf4, but is also involved in the reprogramming of differentiated cells towards the induction of pluripotent stem (iPS) cells (9,10). Several tumor cell types have been reported to express Nanog (11,12). In 2009, You *et al* (13) reported that Nanog was abnormally overexpressed in human embryonic carcinoma NCCIT cells. Downregulation of Nanog by histone deacetylase inhibitor apicidin could lead to cell cycle arrest, differentiation and apoptosis in NCCIT cells. Subsequently, Zbinden *et al* demonstrated the function of Nanog in human glioblastomas and its relationship with HH-GLI activity (14). Our preliminary research has demonstrated overexpression of Nanog in glioma tissues and brain tumor stem cells (BTSCs) compared with normal brain tissues, indicating that Nanog may contribute to the existence of BTSCs and may be related to tumorigenesis of the cerebrum by maintaining the undifferentiated state of glioma cells (15).

MiR-134 is a brain-enriched microRNA which can promote vertebrate central nervous system development (including neuron, cylindraxile and dendrite) (16–19). Reporter assay with 3′-UTR of Nanog cloned downstream of the luciferase gene showed reduced luciferase activity in the presence of miR-134, strongly supporting miR-134 was a direct regulator of Nanog (16). It is worth noting that miR-134 is essential in the promotion of embryonic stem cell differentiation by its direct translational attenuation of Nanog. Data revealed that miR-134 alone can enhance the differentiation of mESCs to ectodermal lineages and modulate mESC (mouse embryonic stem cells) differentiation through its capacity to target and regulate multiple mRNAs, especially Nanog (16).

In this study, we focused on the downregulated miR-134, in human glioma tissues and in glioblastoma cell line U87 then compared it to normal brain tissues, finding that ectopic expression of miR-134 reduced the level of Nanog expression, causing inhibition of proliferation, invasiveness and migration, it also increased apoptosis in glioblastoma cells. These findings suggested that miR-134 could act as a biomarker in glioma and its restoration might be a possible therapeutic approach aimed at Nanog, that deserve further investigation in glioma and BTSCs.

## Materials and methods

### Cell lines and human tissue samples

The U87 glioblastoma cells and 293T cells (Chinese Academy of Sciences Type Culture Collection) were cultured as a monolayer of cells in Dulbecco’s modified Eagle’s medium: nutrient mixture F-12 (Ham’s) (1:1) (DMEM/F-12) (Gibco, USA), supplemented with 10% fetal bovine serum (FBS) (Hyclone, USA), 100 U/ml penicillin/streptomycin (Gibco).

### Clinical sample collection

Glioma samples were obtained from 42 patients with primary gliomas who underwent surgical treatment at the Department of Neurosurgery, Anhui Provincial Hospital Affiliated to Anhui Medial University, between October 2010 and September 2011 in accordance with the national regulation of clinical sampling in China. Eleven adult normal brain tissues as normal controls were obtained from the patients with severe traumatic brain injury (TBI) who needed post-trauma surgery after informed consent. This study was approved by the hospital institutional review board and written informed consent was obtained from all patients. Tumor specimens were immediately sectioned from the resected glioma tissues, frozen in liquid nitrogen and stored at −80°C until RNA/protein extraction. All of the glioma samples were verified by pathological analysis and classified according to the WHO 2007 classification. There were 13 low-grade (WHO grades I and II) and 31 high-grade tumors (WHO grades III and IV). None of the patients had received chemotherapy, immunotherapy and radiotherapy prior to specimen collection. Informed consent was obtained from all patients before surgery as advocated by the regional ethics committee.

### RNA extraction, real-time PCR

Total RNA was extracted from the cultured cells or the human glioma samples with TRIzol reagent (Invitrogen, USA). Relative levels of miR-134 were examined using SYBR green real-time quantitative reverse transcription-PCR (real-time PCR) and normalized with U6 snRNA. cDNA was synthesized by using miScript II RT kit (Qiagen, USA). The primers of miR-134 and U6 snRNA and miScript SYBR Green PCR kit were also purchased from Qiagen for the reaction system of real-time PCR. The real-time PCR reactions were performed on a 7500 Fast System real-time PCR cycler (Applied Biosystems, USA) for 40 cycles. The procedure for PCR was 95°C for 15 min; 94°C for 15 sec, 55°C for 30 sec, 70°C for 30 sec. All procedures were performed according to the instructions provided by the manufacturer. The fold-change of each miRNA was calculated using the 2^ΔΔCt^ method (20).

### RT-PCR

Reverse transcription-polymerase chain reaction (RT-PCR) was performed as described before (15). The primer sequences were as follows: Nanog (403 bp), 5′-ATGCCTGTGATTTGTGGGCC-3′ (forward) and 5′-GCCAGTTGTTTTTCTGCCAC-3′ (reverse); β-actin (252 bp), 5′-ATGGATGATGATATCGCCGCGCTC-3′ (forward) and 5′-TTTCTCCATGTCGTCCCAGTTGG-3′ (reverse). β-actin was used as the internal control.

### Constructs and cell lines

Mature miR-134 sequence (UGUGACUGGUUGACCAGAGGGG) was transformed into premiRNA sequence that could be more suitable for expression. A genomic sequence spanning pre-miR-134 was amplified using primers (miR-134-F: 5′-TGCTGTGTGACTGGTTGACCAGAGGGGGTTTTGGCCACTGACTGACCCCCTCTGCAACCAGTCACA-3′; miR-134-R: 5′-CCTGTGTGACTGGTTGCAGAGGGGGTCAGTCAGTGGCCAAAACCCCCTCTGGTCAACCAGTCACAC-3′) and then cloned into the pLenti6.3/V5-DEST Gateway Vector (Invitrogen); lentiviruses were packaged in HEK293T cells according to the manufacturer’s instructions and were used to infect U87 glioblastoma cells with polybrene (Sigma, USA). Cells were then subcultured to 10% confluence in medium containing 10 *μ*g/ml of blasticidin (Sigma). The process was similar to a previous assay (21). The cells expressing pLenti-miR-134-GFP/pLenti-GFP were termed U87-miR-134/vector-control, respectively, while U87 cells without any treatment were the blank group. The expression of miR-134 was detected by real-time PCR.

### Western blotting

Western blotting and immunohistochemistry assays were performed as previously described (15). Membranes were probed with mouse polyclonal antibodies against human Nanog (1:100 dilution) (R&D Systems, USA) at 4°C overnight or mouse monoclonal anti-β-actin antibody (1:1,000 dilution) (Beyotime, China) for 1 h at room temperature followed by the horseradish peroxidase (HRP)-conjugated goat anti-mouse IgG antibody (ZSGB-BIO, China). Immunoblots were visualized by chemiluminescence using the ECL detection system (BeyoECL Plus, Beyotime). The intensity of the bands was determined using the Image-pro plus 6.0 software (Japan).

### Proliferation assay

MTT assay was used to quantitate cell viability of glioblastoma cells as previously described (22). The absorbance values of each well were measured with a micro-plate spectrophotometer (Molecular Devices, USA) at 570 nm at 24, 48, 72 and 96 h. All proliferation assays were repeated as independent experiments at least three times.

### Tumor growth in vivo

Glioma tumor xenografts were established in female BALB/c athymic mice (Cancer Institute of The Chinese Academy of Medical Science) by subcutaneous injection totals of 5×10^6^ stable U87-miR-134, vector-control cells, while U87 glioblastoma cells as blank group (8 mice per group). All experimental procedures were performed according to Anhui Medical University policies. Tumor growth was measured serially and the tumor volume was measured twice a week with the formula: volume = length × width^2^/2. Paraffin sections of xenograft tumors were subjected to immunohistochemical staining.

### Immunohistochemistry

Immunohistochemical studies were performed as previously described (15). Endogenous peroxidase was neutralized with 3% H_2_O_2_ in methanol (10 min) after antigen retrieval in 0.1 M citrate buffer (pH 5.8) at 95°C for 5 min and cooled at 25°C for 1 h. Sections were blocked with normal goat serum (10 min), then treated with the following primary antibodies overnight at 4°C; NANOG (1:100 dilution). Negative control sections were incubated with PBS instead of the primary antibody. After treatment with biotinylated secondary antibody, color reactions were performed with diaminobenzidine (DAB) (Sigma) and counterstained with Mayer’s hematoxylin. The immunohistochemical staining results were scored by two pathologists.

### Transwell assay and Transwell matrix penetration assay

Cells (4×10^4^) in 200 *μ*l serum-free DMEM were plated on the upper compartment of a Transwell device (without Matrigel for Transwell assay) or plated on the top side of polycarbonate Transwell filter coated with Matrigel (for Transwell matrix penetration assay) in the upper chamber of the BioCoat™ Invasion Chambers (BD, USA) and incubated at 37°C for 24 h, followed by removal of cells inside the upper chamber with cotton swabs. Migrated and invaded cells on the lower membrane surface were fixed in 1% paraformaldehyde, stained with 0.1% crystal violet and counted (8 random 200x fields per well). Cell counts were expressed as the mean number of cells per field of view.

### Wound scraping assay

U87-miR-134 and vector-control cells were plated in 6-well plates and were grown to 80–90% confluence. Then, the monolayer of cells was scraped with a standard 200-*μ*l sterile micropipette tip to create a denuded gap of constant width. The cells were washed with PBS subsequently and then maintain in serum-free medium. After 48 h, the cells migrated into the gap were observed under a phase microscope qualitatively.

### Apoptosis assay

With DAPI staining method, transfected cells were fixed for 20 min in 4% (v/v) paraformaldehyde at 4°C and then incubated with DAPI dye (Sigma) (10 *μ*g/ml) for 15 min. After washing with PBS, cells were observed using an inverted fluorescence microscope (IX70; Olympus, Japan). Transfected cells were detached from culture flask, washed, suspended in PBS and concentrated by centrifugation. Cell samples were fixed in 2.5% (w/v) glutaraldehyde, postfixed in 2% (w/v) buffered osmium tetroxide for 2 h and dehydrated in ethanol. Specimens for transmission electron microscopy were embedded in Epon. Sections were cut using an ultra-microtome and double-stained with uranyl acetate and lead citrate. Electron micrography was performed (JEM-2000EX; Jeol, Japan) using an operating voltage of 80 kV (23). In the flow cytometry assay, transfected cells were dual stained with the viability dye 7-amino-actinomycin D (7AAD) and Annexin V-PE using an Annexin V-PE/7-AAD apoptosis detection kit (KeyGen Biotech, China) according to the manufacturer’s protocol. Stained cells were immediately analysed with a flow cytometer (Cell Lab Quanta SC; Beckman Coulter, USA).

### Statistical analysis

Experimental data are presented as mean ± standard deviation (SD). All statistical analyses were performed using a two-tailed Student’s t-test or one-way ANOVA (SPSS 17.0). P<0.05 was considered statistically significant. All experiments were repeated three times.

## Results

### Downregulation of miR-134 in human glioma tissues and glioblastoma cell line U87

**Figure 1:**
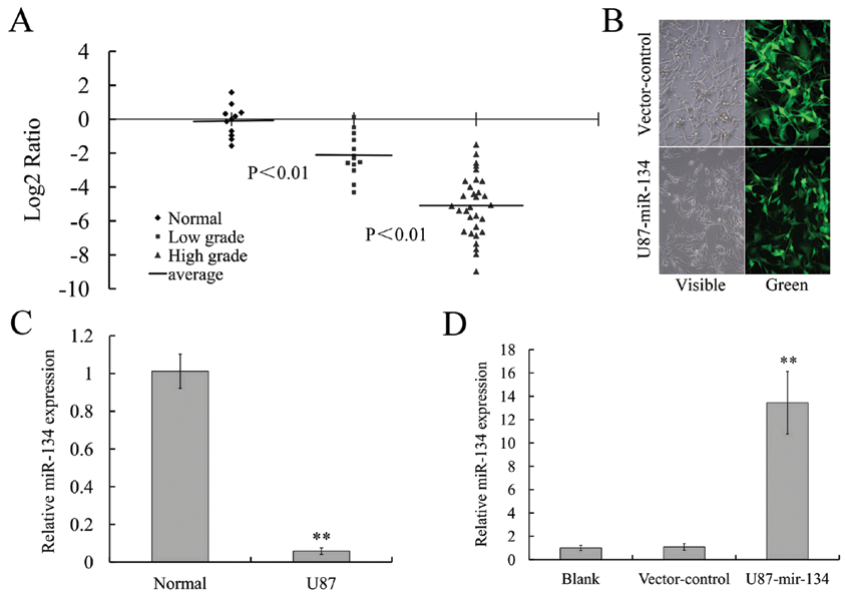
Downregulation of miR-134 in human glioma tissues and glioblastoma cell line U87. (A) Real-time PCR analysis of miR-134 expression in glioma tissues and normal brain tissues. U6 was used as loading control. (C) MiR-134 expression in U87 glioblastoma cells is downregulated compared with normal brain tissues (P<0.01, independent Student’s t-test). (B) Establishment of glioblastoma stable cell lines (magnification, ×400). (D) Real-time PCR analysis of miR-134 expression in glioblastoma stable cell lines (U87-miR-134 and vector-control), compared with U87 cells (blank group), U6 was used as loading control (P<0.01, ANOVA). ^**^P<0.01.

Real-time PCR analysis revealed that the expression of miR-134 was significantly lower in glioma samples compared with normal brain tissues (P<0.01) and was lower in grade III and IV gliomas compared to grade I and II tumors (P<0.01; Fig. 1A). Remarkable downregulation of miR-134 could also be observed in glioblastoma cell line U87 (P<0.01; Fig. 1C). These results suggested that miR-134 could be closely related to human glioma and the low level of miR-134 might be related to glioma oncogenesis and its invasive propensity.

### Ectopic expression of miR-134 reduced the expression of its miRNA target NANOG in U87 glioblastoma cells

MiR-134 expressing the U87 stable cell line was generated for the effect of microRNA-134 on NANOG protein level with lentivirus transfection method. As shown in Fig. 1B, green fluorescent signal was detected in >98% GFP-labeled miR-134-transfected U87 cells, indicating that RNA oligonucleotides could readily gain access to the cells. The expression of miR-134 in U87 cells was detected by real-time PCR. We determined that the expression of miR-134 was significantly increased in U87-miR-134 cells, while in vector-control cells it did not change obviously (Fig. 1D).

**Figure 2:**
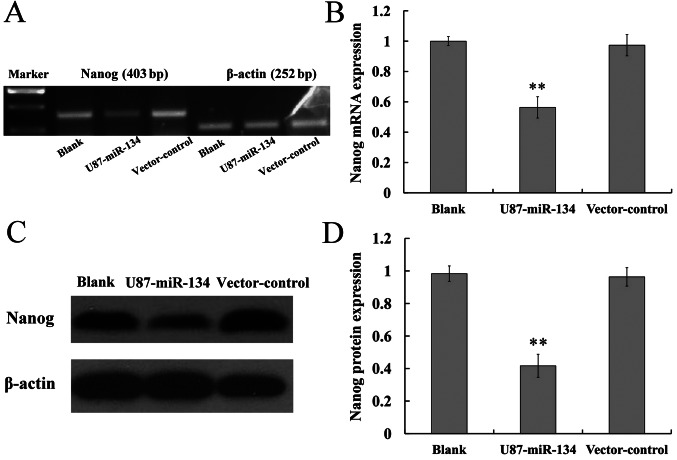
Nanog expression is inhibited by miR-134 in U87 cells. (A) The expression of Nanog in mRNA level was measured by RT-PCR. (B) Histogram representing relative levels of Nanog mRNA by RT-PCR after transducting miR-134 to U87 cells (P<0.01, ANOVA). (C) Western blot analysis of Nanog expression in protein level. (D) Histogram representing the relative level of Nanog protein as determined by western blot analysis (P<0.01, ANOVA). ^**^P<0.01.

Nanog is a comfirmed miR-134 target with 3′-UTR luciferase assays reported by Tay *et al* (16). We therefore determined whether the Nanog mRNA and protein levels could be affected by ectopic expression of miR-134 in glioblastoma cells by RT-PCR and western blotting, respectively. Results revealed that miR-134 can restrain the mRNA and protein expression of Nanog in glioblastoma cells (P<0.01) (Fig. 2). The immunohistochemical staining results (Fig. 5B) also showed that the glioma xenografts of U87-miR-134 group expressed less Nanog than the tumors in the blank and vector-control group (P<0.01).

### MiR-134 affects the invasiveness and migration capability in U87 glioblastoma cells

**Figure 3:**
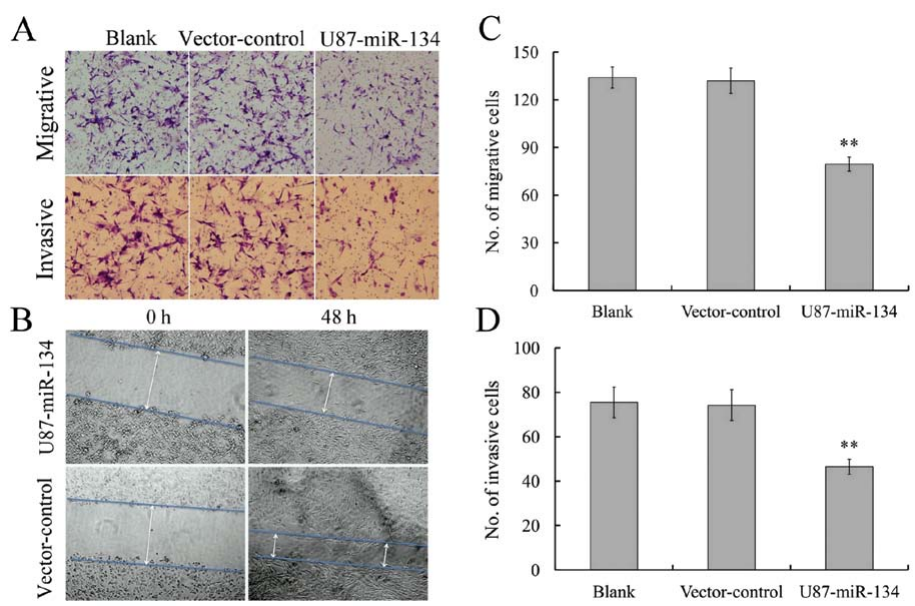
MiR-134 overexpression inhibits the migrative and invasive ability of glioma cells. (A) The number of migrative cells was determined using Transwell assay (without Matrigel). The number of invasive cells was determined using transwell matrix penetration assay (with Matrigel) (magnification, ×200). (B) miR-134 expression inhibited wound closure speed of U87 cells (magnification, ×200). (C) Histogram representing the number of migrative cells per 200x field (P<0.01, ANOVA). (D) Histogram representing the number of invasive cells per 200x field (P<0.01, ANOVA). ^**^P<0.01.

Since invasiveness is one of the pathophysiological features of glioblastoma, we asked whether miR-134 overexpression was associated with the invasiveness of gliomas. Transwell assay (without Matrigel) showed that the migratory speed of U87 glioblastoma cells highly expressing miR-134 was markedly slower than that of control cells (Fig. 3A and C). Furthermore, Transwell matrix penetration (coated with Matrigel) assay showed that the upregulation of miR-134 dramatically reduced the invasiveness of U87 glioblastoma cells (Fig. 3A and D). Wound healing assay showed that miR-134 expression inhibited wound closure speed of U87 cells (Fig. 3B). These findings suggested that miR-134 could substantially inhibit migration and invasion of U87 glioblastoma cells *in vitro*.

### MiR-134 affects the proliferation of U87 glioblastoma cells in vitro and vivo

**Figure 4:**
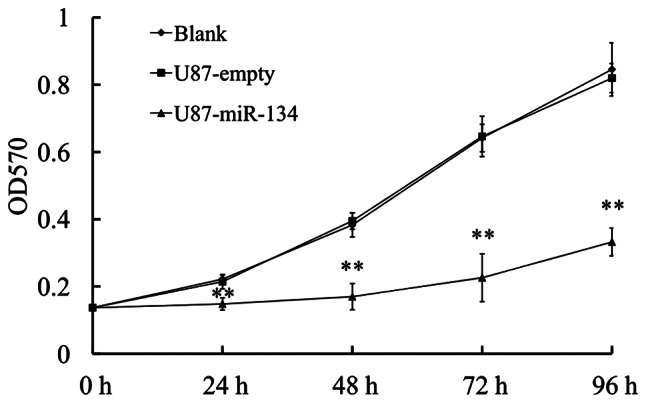
MiR-134 overexpression reduced the proliferation of U87 glioblastoma cells *in vitro* measured by MTT assay. **P<0.01.

We further detected the effect of miR-134 overexpression on the proliferative ability of glioblastoma cells by MTT assay. The result revealed that U87-miR-134 cells showed a significant reduction in cell viability compared to vector-control or the blank group (P<0.01) (Fig. 4).

**Figure 5:**
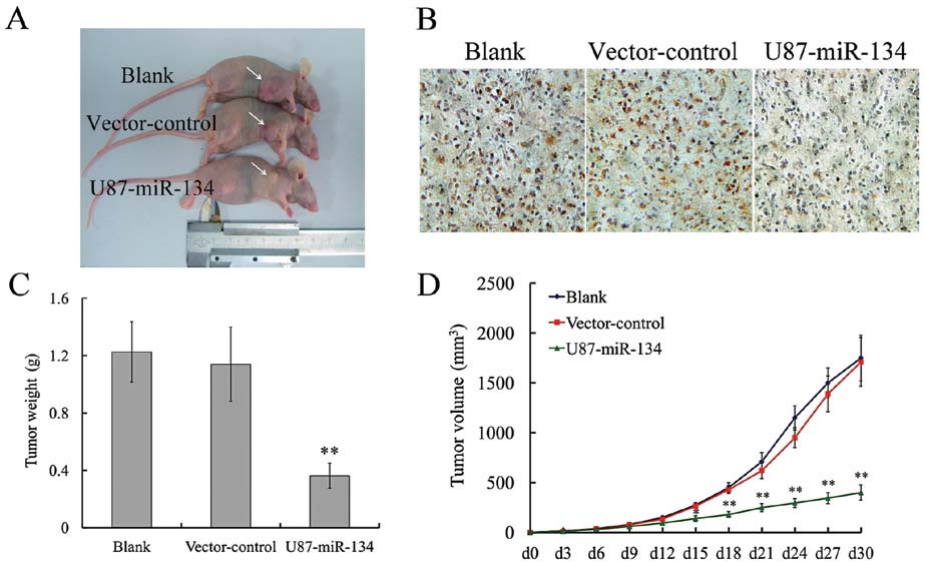
MiR-134 overexpression reduced the proliferation of U87 glioblastoma cells *in vivo*. (A) The establishment of xenograft tumor model. U87-miR-134 glioma tumor xenografts were visibly smaller at the time animals were sacrificed. White arrows indicate glioma tumor xenografts. (B) Nanog protein expression in glioma tumor xenografts were determined by immunohistochemistry assay. (C and D) Tumor mass histograms and tumor growth curves for glioma tumor xenografts (n=8 for each group). ^*^P<0.05; ^**^P<0.01.

To confirm the effect of miR-134 *in vivo*, tumor xenograft animal model was performed. Thirty days after subcutaneous inoculation of U87-miR-134, vector-control or blank group cells into nude mice, each cell inoculation developed into solid tumor xenografts (Fig. 5). The growth curve of tumor xenografts showed that high miR-134 level significantly slowed down tumor growth *in vivo*, while there was no statistical difference between vector-control and blank groups (Fig. 5D). The experimental mice were euthanized on day 36. The average tumor weight in the vector-control group was significantly higher than in the U87-miR-134 group (Fig. 5C). These findings indicated that exogenous miR-134 was able to inhibit glioma growth *in vitro* and *in vivo*.

### MiR-134 promotes apoptosis in U87 glioblastoma cells

**Figure 6:**
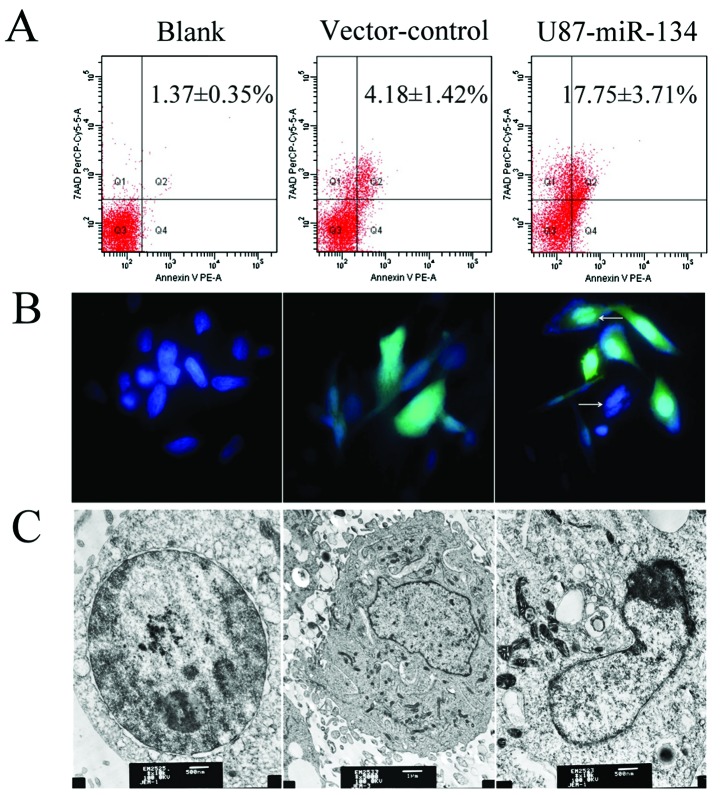
MiR-134 overexpression induced apoptosis in U87 glioblastoma cells. (A) The percentage of apoptotic cells were determined by flow cytometry. (B) DAPI staining. White arrows indicate apoptotic cells in U87-miR-134 cells. Original magnification, ×400. (C) Transmission electron microscopy. Left, blank cells; original magnification, ×10,000; bar, 500 nm; middle, vector-control cells; original magnification, ×5,000; bar, 1 *μ*m; right, U87-miR-134 cells; original magnification, ×10,000; bar, 500 nm.

Nanog knockdown induced cell cycle arrest, and apoptosis, whereas suppressed proliferation and telomerase activity in mESCs (24). To confirm the effect of miR-134 overexpression in U87 glioblastoma cell, flow cytometry, DAPI staining and transmission electron microscopy methods were used. Phosphatidylserine externalization was assayed by flow cytometry using Annexin V-PE/7-amino-actinomycin D double-stained U87 cells. As shown in Fig. 6A, the percentage of the apoptotic cells were significantly higher in U87-miR-134 (17.75±3.71%) compared with the vector-control (4.18±1.42%) and blank group (1.37±0.35%) (P<0.01, ANOVA). Typical apoptotic morphological changes were found in U87-miR-134 cells including shrinkage, deformation and detachment from culture dishes. Nuclear condensation and chromatin margination were observed using an inverted fluorescence microscope (Fig. 6B) and chromatin margination, nuclear condensation were noted under transmission electron microscopy (Fig. 6C). These statistical results and morphological changes elucidated the apoptotic inducing ability of miR-134 to repress tumor proliferation.

## Discussion

Accumulated evidence shows that miRNAs have an important impact on tumor gene expression and it might play a key role in tumorigenesis due to their widespread dysregulation. MiR-134 is located in a very large cluster of brain-specific miRNAs at chromosome 14q32.31 (25). It is worth noting that miR-134 is detected in low expression in oligodendrogliomas (ODG) and glioblastomas (GBM) (26). Whereas Nanog, which could be post-transcriptionally downregulated by miR-134, is upregulated in glioma tissues and BTSCs (14,15). However, the association between pathological grade of glioma and miR-134 expression, downregulation of Nanog expression by miR134 in glioblastoma cells and knockdown of Nanog impacting the proliferation and progression of glioblastoma cells are still unclear.

In the present study, we investigated the expression of miR-134 in glioma tissues and U87 glioblastoma cells and found miR-134 was downregulated in glioma and U87 cell line compared to normal brain tissues. It is noteworthy that miR-134 expression was significantly lower in grade III and IV glioma compared to grade I and II tumors. It was suggested that miR-134 might be a novel specific biomarker for gliomas. In addition the loss of miR-134 could be involved in glioma development. The miR-134 ectopic expression slowed down tumor cells growth *in vitro* and *in vivo*, compared to vector-control and blank groups. Furthermore, miR-134 overexpression *in vitro* inhibited migration and invasion and significantly increased apoptosis in U87 glioblastoma cells. We suggest that miR-134 may play a critical role in brain tumorigenesis and progression.

Nanog overexpression has already been detected in a number of human tumors, including glioma cells and it participated in some oncogenic pathways (11–14). Nanog, Stat-3 and miR-21 could form a functional signaling axis that provides therapeutic targets to cause tumor cell apoptosis and overcome cisplatin chemoresistance (12,27). Moreover, Nanog and GLI1 are able to form a positive functional loop modulated by p53; Nanog expression depends on endogenous HH-GLI activity and its function is required for glioblastoma growth *in vivo* (14). We further confirmed that Nanog mRNA and protein expressions were substantially downregulated by ectopic miR-134 in U87 glioblastoma cells. Our tumor growth assays also indicated that Nanog was associated with GBM tumorigenicity *in vivo*. However, whether miR-134 can function independently of Nanog and if miR-134 has other targets that affect glioma cell growth are still unknown. Therefore, further research aimed at Nanog is needed for glioma carcinogenesis.

In conclusion, comprehensive analysis indicated that loss of miR-134 expression is a common event in glioma and miR-134 overexpression reduced the proliferation, invasiveness and migration capabilities. MiR-134 overexpression also promoted apoptosis in glioblastoma cells. Restoration of miR-134 expression may represent a novel therapeutic approach in multi-modal therapy for poorly differentiated glioblastoma. However, more investigations are required to determine the possible promising roles of miR-134 and Nanog in Nanog-Stat-3-miR-21 signaling axis and functional Gli-Nanog-P53 network (14,28). Further testing of miR-134 in preclinical models of glioblastoma in conjunction with various delivery strategies will help define its ultimate therapeutic potential for treatment of glioblastoma.
